# Structural determination of *Rickettsia* lipid A without chemical extraction confirms shorter acyl chains in later-evolving spotted fever group pathogens

**DOI:** 10.1128/msphere.00609-23

**Published:** 2024-01-23

**Authors:** Hyojik Yang, Victoria I. Verhoeve, Courtney E. Chandler, Shreeram Nallar, Greg A. Snyder, Robert K. Ernst, Joseph J. Gillespie

**Affiliations:** 1 Department of Microbial Pathogenesis, School of Dentistry, University of Maryland, Baltimore, Maryland, USA; 2 Department of Microbiology and Immunology, School of Medicine, University of Maryland, Baltimore, Maryland, USA; 3 Division of Vaccine Research, Institute of Human Virology, University of Maryland, Baltimore, Maryland, USA; The University of Texas Medical Branch at Galveston, Galveston, Texas, USA

**Keywords:** *Rickettsia*, lipopolysaccharide, lipid A, FLAT^n^, pathogenesis, rickettsiosis, spotted fever group, evolution, Rocky Mountain spotted fever

## Abstract

Rickettsiae are Gram-negative obligate intracellular parasites of numerous eukaryotes. Human pathogens of the transitional group (TRG), typhus group (TG), and spotted fever group (SFG) rickettsiae infect blood-feeding arthropods, have dissimilar clinical manifestations, and possess unique genomic and morphological attributes. Lacking glycolysis, rickettsiae pilfer numerous metabolites from the host cytosol to synthesize peptidoglycan and lipopolysaccharide (LPS). For LPS, O-antigen immunogenicity varies between SFG and TG pathogens; however, lipid A proinflammatory potential is unknown. We previously demonstrated that *Rickettsia akari* (TRG), *Rickettsia typhi* (TG), and *Rickettsia montanensis* (SFG) produce lipid A with long 2′ secondary acyl chains (C16 or C18) compared to short 2′ secondary acyl chains (C12) in *Rickettsia rickettsii* (SFG) lipid A. To further probe this structural heterogeneity and estimate a time point when shorter 2′ secondary acyl chains originated, we generated lipid A structures for two additional SFG rickettsiae (*Rickettsia rhipicephali* and *Rickettsia parkeri*) utilizing fast lipid analysis technique adopted for use with tandem mass spectrometry (FLAT^n^). FLAT^n^ allowed analysis of lipid A structure directly from host cell-purified bacteria, providing a substantial improvement over lipid A chemical extraction. FLAT^n^-derived structures indicate SFG rickettsiae diverging after *R. rhipicephali* evolved shorter 2′ secondary acyl chains. While 2′ secondary acyl chain lengths do not distinguish *Rickettsia* pathogens from non-pathogens, *in silico* analyses of *Rickettsia* LpxL late acyltransferases revealed discrete active sites and hydrocarbon rulers for long versus short 2′ secondary acyl chain addition. Our collective data warrant determining *Rickettsia* lipid A inflammatory potential and how structural heterogeneity impacts lipid A-host receptor interactions.

**IMPORTANCE:**

Deforestation, urbanization, and homelessness lead to spikes in Rickettsioses. Vector-borne human pathogens of transitional group (TRG), typhus group (TG), and spotted fever group (SFG) rickettsiae differ by clinical manifestations, immunopathology, genome composition, and morphology. We previously showed that lipid A (or endotoxin), the membrane anchor of Gram-negative bacterial lipopolysaccharide (LPS), structurally differs in *Rickettsia rickettsii* (later-evolving SFG) relative to *Rickettsia montanensis* (basal SFG), *Rickettsia typhi* (TG), and *Rickettsia akari* (TRG). As lipid A structure influences recognition potential in vertebrate LPS sensors, further assessment of *Rickettsia* lipid A structural heterogeneity is needed. Here, we sidestepped the difficulty of *ex vivo* lipid A chemical extraction by utilizing fast lipid analysis technique adopted for use with tandem mass spectrometry, a new procedure for generating lipid A structures directly from host cell-purified bacteria. These data confirm that later-evolving SFG pathogens synthesize structurally distinct lipid A. Our findings impact interpreting immune responses to different *Rickettsia* pathogens and utilizing lipid A adjuvant or anti-inflammatory properties in vaccinology.

## OBSERVATION

Genus *Rickettsia* (*Alphaproteobacteria*: Rickettsiales) comprises species of obligate intracellular parasites of numerous eukaryotes (1). Across the *Rickettsia* phylogeny, all known agents of human disease are vector-borne pathogens of the transitional group (TRG), typhus group (TG), or spotted fever group (SFG) rickettsiae, which are later-evolving clades relative to basal lineages of numerous invertebrate and protist endosymbionts (2). Compared to other Rickettsiales species with human health relevance (e.g., *Orientia tsutsugamushi*, *Wolbachia*, species of *Neorickettsia*, *Anaplasma*, *Neoehrlichia*, and *Ehrlichia*), rickettsiae alone synthesize lipopolysaccharide (LPS) (3), which comprises extracellular polysaccharide chains (O-antigen) linked to a membrane phosphoglycolipid (lipid A) by a core oligosaccharide. As all *Rickettsia* species lack glycolytic enzymes, they are the only known bacteria to synthesize a canonical Gram-negative cell envelope rich in LPS, as well as peptidoglycan (4, 5), from metabolites derived from host cytosol (6).

All described rickettsioses are caused by arthropod-borne pathogens that differ in their protein secretome (7, 8) and presumably LPS composition. For LPS, *Rickettsia* O-antigen contains the sugar quinovosamine (9–11) that is required for S-layer formation and vertebrate pathogenicity (12). While the proinflammatory nature of *Rickettsia* lipid A remains unknown, it is a candidate for the well-characterized triggering of mammalian MD-2/TLR4 receptor and non-canonical inflammasome activation during infection (13–18). We previously demonstrated that human pathogens *Rickettsia akari* (TRG) and *Rickettsia typhi* (TG), as well as the non-pathogen *Rickettsia montanensis* (SFG), produce lipid A with longer 2′’ secondary acyl chains (C16 or C18) relative to shorter chains (C12) in the pathogen *Rickettsia rickettsii* (SFG) lipid A (19). As *R. rickettsii* is later evolving in the SFG rickettsiae relative to *R. montanensis*, we surmised that a switch from longer to shorter 2′ secondary acyl chains occurred later in SFG rickettsial evolution. This later-evolving clade is dominated by other notable human pathogens, including the agents of Japanese spotted fever (*Rickettsia japonica*), Flinders Island spotted fever (*Rickettsia honei*), Pacific Coast tick fever (*Rickettsia philipii*), Mediterranean spotted fever (*Rickettsia conorii*), Siberian tick typhus (*Rickettsia sibirica*), African tick-bite fever (*Rickettsia africae*), and *Dermacentor*-borne necrosis erythema and lymphadenopathy (*Rickettsia raoultii* and *Rickettsia slovaca*). However, the occurrence of other non-pathogens in this clade (e.g., *Rickettsia peacockii* and the seal fur louse endosymbiont) and uncertain vertebrate infectivity dynamics for many *Rickettsia* species raise questions as to the nature of *Rickettsia* lipid A and its impact on host cell immune systems.

To further evaluate *Rickettsia* lipid A structural heterogeneity, we selected two additional species, *Rickettsia rhipicephali* and *Rickettsia parkeri*, for lipid A structural analysis. In recent phylogeny estimations, *R. rhipicephali* groups in a clade that diverges after *R. montanensis* (long 2′ secondary acyl chains) but before *R. rickettsii* (short 2′ secondary acyl chain), while *R. parkeri* belongs to a clade that is sister to the *R. rickettsii*-containing clade (1, 7). Thus, our strategy zeros in on the evolutionary timepoint for the transition to short 2′ secondary acyl chains in SFG rickettsiae. Furthermore, we utilized a different analytical approach to generate new structures called fast lipid analysis technique adopted for use with tandem mass spectrometry (FLAT^n^), allowing assessment of prior structure determinations that were based on lipid A chemical extraction. Briefly, FLAT^n^ is a method for the on-surface and/or on-tissue release of lipid A from LPS that allows its detection by matrix-assisted laser desorption ionization mass spectrometry in the negative ion mode (20, 21) and has been shown to facilitate direct analysis of lipid A structure from a single bacteria colony (22). Therefore, FLAT^n^ circumvents time-, labor-, and sample-intensive techniques previously required to chemically extract lipid A prior to MS analysis. Thus, we reasoned that FLAT^n^ would yield *Rickettsia* lipid A structures from a minimal sample of bacteria partially purified from far fewer host cells than our prior approach.

**Fig 1:**
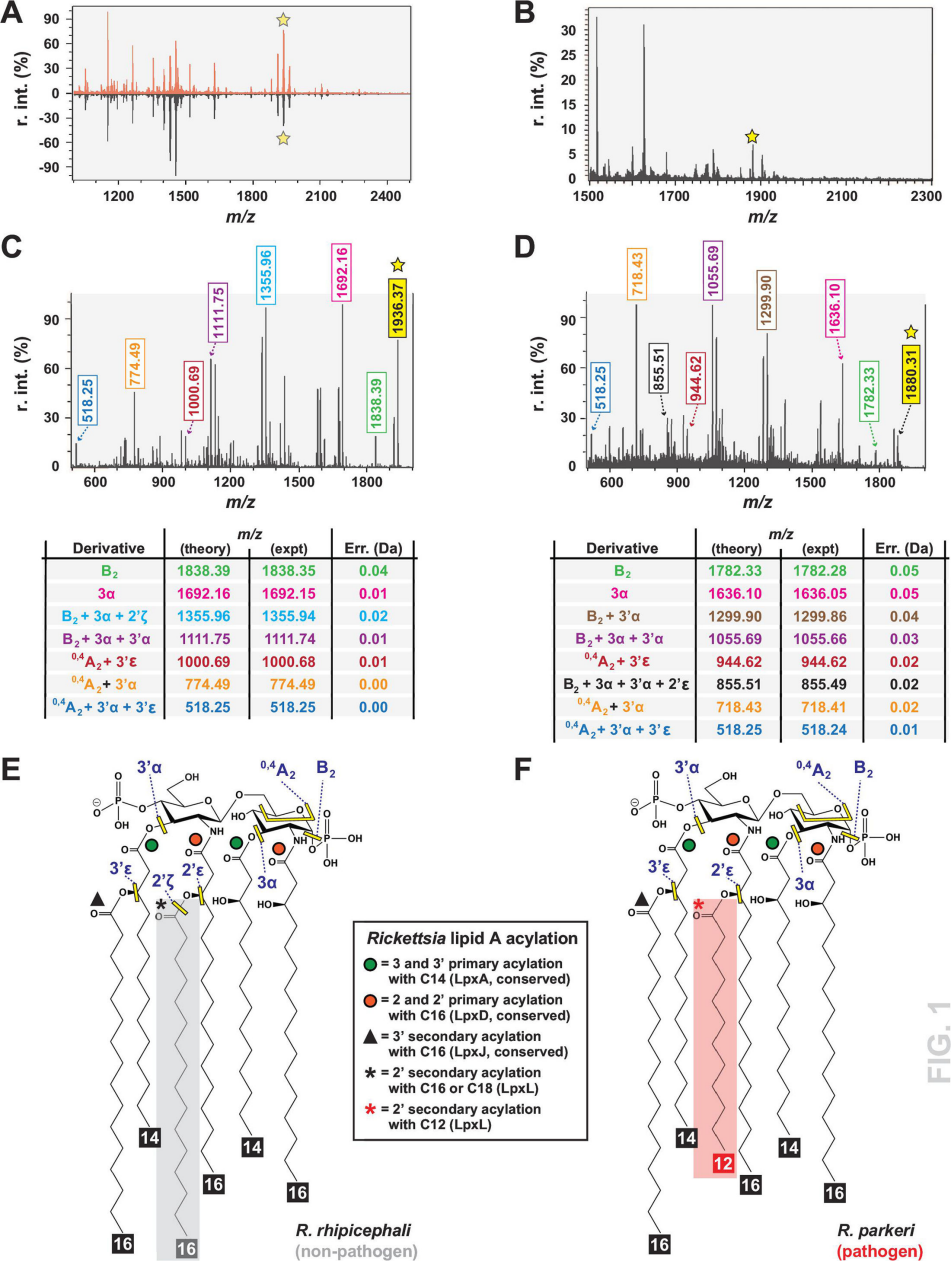
*Rickettsia* lipid A structures determined by FLAT^n^ confirm variable acyl chain lengths for different rickettsiae. (**A and B**) FLAT-MS spectra from (**A**) *R. rhipicephali* partially purified from host cells using either a bead (**B**) or sucrose gradient (S) purification strategy, and from (**B**) *R. parkeri* partially purified from host cells using sucrose gradient purification strategy. Briefly, Vero76 cells were grown to confluence in T-25 flasks with cells infected at an MOI of 10. At 3 days post-infection, host cells were recovered and lysed with 3 mm beads, host debris was removed via low-speed centrifugation (5,000 rpm), and *Rickettsia* was collected by high-speed centrifugation (8,000 rpm). Purification via sucrose gradient followed our prior protocol (19). Bacterial pellets were then analyzed via FLAT^n^. Asterisks indicate the expected size for *Rickettsia* lipid A with C16 (~1,936.37 *m*/*z*) or C12 (1,880.31 *m*/*z*) 2′ secondary acyl chains based on our prior report (19). (**C and D**) Derivatization of a single ion for the (**C**) *R. rhipicephali* sample (1,936.37 *m*/*z*) and the (**D**) *R. parkeri* sample illustrating five and six, respectively, major fragmentation products. These products are named in the tables with theoretical and experimental sizes shown, with error calculation illustrating robust interpretation. They are also color-coded to facilitate the interpretation of the spectra above and predicted structures. (**E and F**) FLAT^n^-derived structure predictions for lipid A of (**E**) *R. rhipicephali*, which is similar to previously determined *R. akari*, *R. typhi,* and *R. montanensis* structures, and (**F**) *R. parkeri*, which is similar to previously determined structures for *R. rickettsii* strains Shelia Smith and Iowa. Sites yielding fragmentation products are yellow, with corresponding nomenclature described in the tables in panels **C** and **D**. The inset describes the conserved and variable lipid A acylation of *Rickettsia* lipid A, with colored symbols mapped on structures (see Fig. S1 for more details).

FLAT^n^ allowed for the direct analysis of *R. rhipicephali* and *R. parkeri* lipid A structures from host cells, providing substantial improvement and efficiency over lipid A chemical extraction (Fig. 1). Bacterial samples that were purified from host cells using either bead- or sucrose gradient-based strategies sufficed to generate MS spectra at either 1,936.37 *m/z* (C16 2′ secondary acyl chains) or 1,880.31 *m/z* (C12 2′ secondary acyl chains), consistent with prior analyses of chemically extracted *Rickettsia* lipid A (19) (Fig. 1A and B). Subsequent derivatization of these single ions for *R. rhipicephali* (Fig. 1C) and *R. parkeri* (Fig. 1D) yielded fragmentation products that supported structural elucidation. The FLAT^n^-derived structure for *R. rhipicephali* lipid A corroborates those of *R. akari*, *R. typhi*, and *R. montanensis* with long 2′ secondary acyl chains (Fig. 1E). In contrast, the *R. parkeri* FLAT^n^-derived lipid A structure matches that determined for *R. rickettsii* strains with short 2′ secondary acyl chains (Fig. 1F). Thus, as with prior results (19), we have discovered distinct lipid A in later-evolving SFG rickettsiae that is more structurally similar to inflammatory lipid A of certain Enterobacteriaceae species than other *Rickettsia* lipid A with longer 2′ secondary acyl chains.

**Fig 2:**
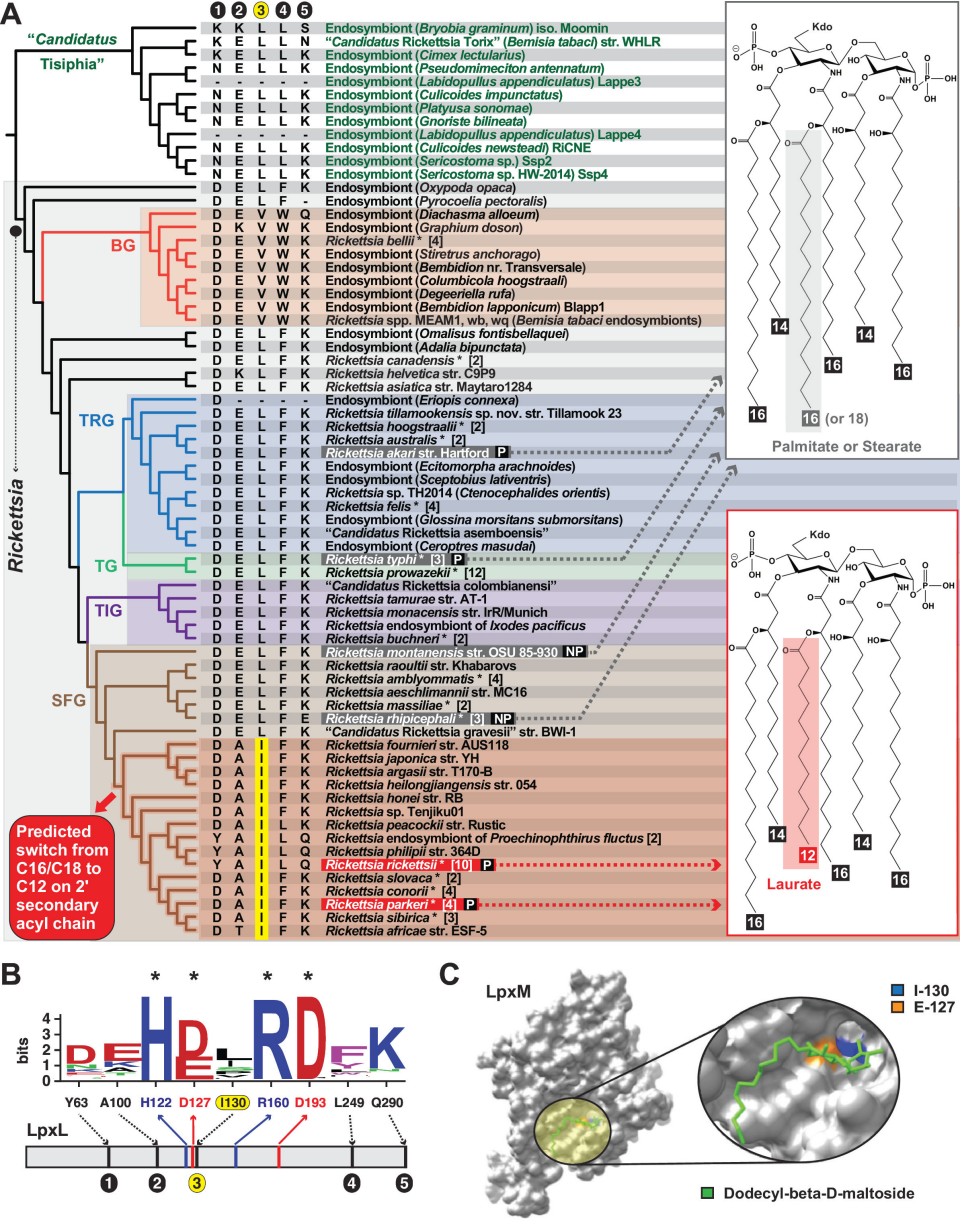
The evolution of variable acyl chain lengths in *Rickettsia* lipid A. (**A**) Superimposition of determined lipid A structures and late acyltransferase LpxL characteristics over an estimated *Rickettsia* phylogeny. Tree is redrawn from a recent study (7). BG, Bellii group and TIG, Tamurae/Ixodes group. Taxon names in gray boxes were determined to synthesize lipid A with palmitate or stearate (C16 or C18) added to the primary 2′ acyl chain; taxon names in red boxes were determined to synthesize lipid A with palmitate or stearate (C16 or C18) added to the primary 2′ acyl chain (see Fig. S1 for more details). P, human pathogen; NP, non-pathogen. Red shading indicates the predicted time point in SFG rickettsiae evolution where a switch from palmitate/stearate to laurate on lipid A 2′ hydroxypalmitate could have occurred based on our structural determinations and shared features of LpxL proteins. Circles 1–5 depict the only variable positions from an alignment of LpxL proteins (panel **B**). Yellow highlighting indicates a conserved Ile in variable position 3 for the clade-containing species adding laurate on lipid A 2′ hydroxypalmitate. (**B**) Sequence logo (26) showing conservation of acyltransferase active site residues (asterisks) and five variable positions from an alignment of 127 non-redundant rickettsial LpxL proteins. Alignment was performed using MUSCLE (default parameters) (27). Complete information for all proteins is provided in Table S1. Amino acid coloring scheme and assignment are as follows: black, hydrophobic; red, negatively charged; green, hydrophilic; purple, aromatic; and blue, positively charged. The five variable positions are shown for taxa in the estimated phylogeny in panel A. (**C**) Visualization of LpxL variable position 3 within the active site of the lipid A late acyltransferase LpxM (note: LpxM is analogous to *Rickettsia* LpxJ (28)and acylates 3′ acyl chains but was used since no 2′ secondary acyltransferase structures exist). Gray, surface representation of lipid A acyltransferase LpxM from *Acinetobacter baumannii* (PDBID: 5KN7) is shown in gray. Yellow circle highlighting and insert depict n-dodecyl-β-D-maltoside ligand in green. The acidic active site position (E-127, orange) has a reduced substrate binding when mutated to Ala. *Rickettsia* variable position 3 (I-130, blue) is found on the same helix near the catalytic E-127 and may be involved in mediating acyl chain length substrate selectivity. Note: 5KN7-N-130 is mutated to I-130 for this depiction; 5KNK is E-127A mutant structure (not shown). Structures are visualized using Chimera (29).

While bolstering prior results that indicated *Rickettsia* lipid A structural heterogeneity, FLAT^n^-derived structures also helped refine the evolutionary time point when short 2′ secondary acyl chains originated within SFG rickettsiae (Fig. 2A). In light of phylogeny estimation, our collective data indicate SFG rickettsiae diverging after *R. rhipicephali* evolved shorter 2′ secondary acyl chains (Fig. 2A, red shading). A parsimonious interpretation entails all members of the *R. rhipicephali*-containing clade synthesize lipid A with long 2′ secondary acyl chains, whereas all later-evolving SFG rickettsiae synthesize lipid A with short 2′ secondary acyl chains. Aside from many human pathogens in the later-evolving lineages, *R. peacockii* and the endosymbiont of the seal fur louse (*Proechinophthirus fluctus*) are non-pathogenic species that do not infect vertebrates (23, 24). Assuming that these endosymbionts synthesize lipid A [both of their genomes encode the full suite of Raetz pathway enzymes (3)], short 2′ secondary acyl chains could be considered a trait that emerged without selective pressure from the vertebrate host environment. Alternatively, the non-pathogenic endosymbionts of this clade may have lost the ability to invade vertebrate hosts due to the pseudogenization of numerous genes implicated in vertebrate cell invasion (23, 24), including uncharacterized LPS-modification enzymes that may only be necessary for survival in vertebrate hosts (25). Generating lipid A structures for other species in this clade will be necessary to further evaluate our observations, as will determining if rickettsiae alter their lipid A acyl chain lengths in vertebrate versus invertebrate hosts.

Finally, we inspected *Rickettsia* LpxL proteins for possible active site traits that correlate with lipid A structural heterogeneity (Fig. 2A and B). LpxL is the late acyltransferase in the Raetz pathway that acylates 2′ primary acyl chains (see Fig. S1). Of only five variable residues across *Rickettsia* LpxL proteins, two (positions 100 and 130) have distinct properties defining later-evolving SFG rickettsiae (Fig. 2A and B). While nonetheless interesting with short chain (Ala/Thr) replacing charged (Lys/Glu) residues, position 100 is outside of the LpxL active site. However, position 130 (highlighted yellow in Fig. 2A and B) has a conserved Iso in place of Val/Leu and is proximal to active site residue Glu-127, which has been implicated in substrate binding for the analogous late acyltransferase LpxM (30) (Fig. 2C). This compelling correlation of acyl chain length and active site composition across *Rickettsia* phylogeny warrants characterizing substrate specificities for divergent *Rickettsia* LpxL enzymes.

In summary, our work (i) bolsters *Rickettsia* lipid A structural heterogeneity, (ii) shows FLAT^n^ is effective for analyzing obligate intracellular bacterial lipid A, and (iii) identifies LpxL properties that may explain variable acyl chain length addition. We anticipate our findings to impact studies comparing host immune responses to divergent pathogens and inform on the efficacy of lipid A in *Rickettsia* vaccinology.

## SUPPLEMENTAL MATERIAL

Fig. S1 msphere.00609-23-s0001.pdf The Raetz pathway for *Rickettsia* lipid A biosynthesis is variable for 2′ secondary acyl chain addition.

Table S1 msphere.00609-23-s0002.xlsx Rickettsial LpxL sequences used for bioinformatics analyses.

ASM does not own the copyrights to Supplemental Material that may be linked to, or accessed through, an article. The authors have granted ASM a non-exclusive, world-wide license to publish the Supplemental Material files. Please contact the corresponding author directly for reuse.

## References

[1] Davison HR, Pilgrim J, Wybouw N, Parker J, Pirro S, Hunter-Barnett S, Campbell PM, Blow F, Darby AC, Hurst GDD, Siozios S. 2022. Genomic diversity across the Rickettsia and ‘candidatus megaira’ genera and proposal of genus status for the torix group. Nat Commun 13:2630. doi:10.1038/s41467-022-30385-6 35551207 PMC9098888

[2] Pilgrim J, Thongprem P, Davison HR, Siozios S, Baylis M, Zakharov EV, Ratnasingham S, deWaard JR, Macadam CR, Smith MA, Hurst GDD. 2021. Torix Rickettsia are widespread in arthropods and reflect a neglected symbiosis. Gigascience 10:giab021. doi:10.1093/gigascience/giab021 33764469 PMC7992394

[3] Driscoll TP, Verhoeve VI, Guillotte ML, Lehman SS, Rennoll SA, Beier-Sexton M, Rahman MS, Azad AF, Gillespie JJ, Rikihisa Y. 2017. Wholly Rickettsia ! reconstructed metabolic profile of the quintessential bacterial parasite of eukaryotic cells. mBio 8:e00859-17. doi:10.1128/mBio.00859-17 28951473 PMC5615194

[4] Atwal S, Chuenklin S, Bonder EM, Flores J, Gillespie JJ, Driscoll TP, Salje J. 2021. Discovery of a diverse set of bacteria that build their cell walls without the canonical peptidoglycan polymerase apbp. mBio 12:e0134221. doi:10.1128/mBio.01342-21 34311584 PMC8406291

[5] Figueroa-Cuilan WM, Irazoki O, Feeley M, Smith E, Nguyen T, Cava F, Goley ED. 2023. Quantitative analysis of morphogenesis and growth dynamics in an obligate intracellular bacterium. Mol Biol Cell 34:ar69. doi:10.1091/mbc.E23-01-0023 37017481 PMC10295487

[6] Gillespie JJ, Salje J. 2023. Orientia and Rickettsia: different flowers from the same garden. Curr Opin Microbiol 74:102318. doi:10.1016/j.mib.2023.102318 37080115

[7] Verhoeve VI, Lehman SS, Driscoll TP, Beckmann JF, Gillespie JJ. 2023. Metagenome diversity Illuminates origins of pathogen effectors. mBio Revis:2023.02.26.530123. doi:10.1101/2023.02.26.530123 PMC11077975 38564675

[8] Gillespie JJ, Kaur SJ, Rahman MS, Rennoll-Bankert K, Sears KT, Beier-Sexton M, Azad AF. 2015. Secretome of obligate intracellular Rickettsia. FEMS Microbiol Rev 39:47–80. doi:10.1111/1574-6976.12084 25168200 PMC4344940

[9] Amano K, Fujita M, Suto T. 1993. Chemical properties of lipopolysaccharides from spotted fever group Rickettsiae and their common antigenicity with lipopolysaccharides from proteus species. Infect Immun 61:4350–4355. doi:10.1128/iai.61.10.4350-4355.1993 8406824 PMC281165

[10] Amano KI, Williams JC, Dasch GA. 1998. Structural properties of lipopolysaccharides from Rickettsia typhi and Rickettsia prowazekii and their chemical similarity to the lipopolysaccharide from proteus vulgaris OX19 used in the weil-felix test. Infect Immun 66:923–926. doi:10.1128/IAI.66.3.923-926.1998 9488376 PMC107996

[11] Peturova M, Vitiazeva V, Toman R. 2015. Structural features of the O-antigen of Rickettsia typhi, the etiological agent of endemic typhus. Acta Virol 59:228–233. doi:10.4149/av_2015_03_228 26435145

[12] Kim HK, Premaratna R, Missiakas DM, Schneewind O. 2019. Rickettsia conorii O antigen is the target of bactericidal weil-felix antibodies. Proc Natl Acad Sci U S A 116:19659–19664. doi:10.1073/pnas.1911922116 31413191 PMC6765297

[13] Quevedo-Diaz MA, Song C, Xiong Y, Chen H, Wahl LM, Radulovic S, Medvedev AE. 2010. Involvement of TLR2 and TLR4 in cell responses to Rickettsia akari. J Leukoc Biol 88:675–685. doi:10.1189/jlb.1009674 20616112 PMC2974430

[14] Jordan JM, Woods ME, Olano J, Walker DH. 2008. The absence of toll-like receptor 4 signaling in C3H/HeJ mice predisposes them to overwhelming rickettsial infection and decreased protective Th1 responses. Infect Immun 76:3717–3724. doi:10.1128/IAI.00311-08 18490467 PMC2493240

[15] Bechelli J, Smalley C, Zhao X, Judy B, Valdes P, Walker DH, Fang R. 2016. MyD88 mediates instructive signaling in dendritic cells and protective inflammatory response during rickettsial infection. Infect Immun 84:883–893. doi:10.1128/IAI.01361-15 26755162 PMC4807495

[16] Jordan JM, Woods ME, Soong L, Walker DH. 2009. Rickettsiae stimulate dendritic cells through toll-like receptor 4, leading to enhanced NK cell activation in vivo. J Infect Dis 199:236–242. doi:10.1086/595833 19072551 PMC2613164

[17] Smalley C, Bechelli J, Rockx-Brouwer D, Saito T, Azar SR, Ismail N, Walker DH, Fang R, Winslow GM. 2016. Rickettsia australis activates inflammasome in human and murine macrophages. PLoS ONE 11:e0157231. doi:10.1371/journal.pone.0157231 27362650 PMC4928923

[18] Burke TP, Engström P, Chavez RA, Fonbuena JA, Vance RE, Welch MD. 2020. Inflammasome-mediated antagonism of type I interferon enhances Rickettsia pathogenesis. Nat Microbiol 5:688–696. doi:10.1038/s41564-020-0673-5 32123346 PMC7239376

[19] Guillotte ML, Chandler CE, Verhoeve VI, Gillespie JJ, Driscoll TP, Rahman MS, Ernst RK, Azad AF. 2021. Lipid A structural divergence in Rickettsia pathogens. mSphere 6:e00184-21. doi:10.1128/mSphere.00184-21 33952661 PMC8103985

[20] Sorensen M, Chandler CE, Gardner FM, Ramadan S, Khot PD, Leung LM, Farrance CE, Goodlett DR, Ernst RK, Nilsson E. 2020. Rapid microbial identification and colistin resistance detection via MALDI-TOF MS using a novel on-target extraction of membrane lipids. Sci Rep 10:21536. doi:10.1038/s41598-020-78401-3 33299017 PMC7725828

[21] Yang H, Chandler CE, Jackson SN, Woods AS, Goodlett DR, Ernst RK, Scott AJ. 2020. On-tissue derivatization of lipopolysaccharide for detection of lipid A using MALDI-MSI. Anal Chem 92:13667–13671. doi:10.1021/acs.analchem.0c02566 32902263 PMC8717242

[22] Yang H, Smith RD, Chandler CE, Johnson JK, Jackson SN, Woods AS, Scott AJ, Goodlett DR, Ernst RK. 2022. Lipid A structural determination from a single colony. Anal Chem 94:7460–7465. doi:10.1021/acs.analchem.1c05394 35576511 PMC9392460

[23] Felsheim RF, Kurtti TJ, Munderloh UG. 2009. Genome sequence of the endosymbiont Rickettsia peacockii and comparison with virulent Rickettsia rickettsii: identification of virulence factors. PLoS One 4:e8361. doi:10.1371/journal.pone.0008361 20027221 PMC2791219

[24] Boyd BM, Allen JM, Koga R, Fukatsu T, Sweet AD, Johnson KP, Reed DL. 2016. Two bacterial genera, sodalis and Rickettsia, associated with the seal louse proechinophthirus fluctus. Appl Environ Microbiol 82:3185–3197. doi:10.1128/AEM.00282-16 26994086 PMC4959230

[25] Gillespie JJ, Joardar V, Williams KP, Driscoll TP, Hostetler JB, Nordberg E, Shukla M, Walenz B, Hill CA, Nene VM, Azad AF, Sobral BW, Caler E. 2012. A Rickettsia genome overrun by mobile genetic elements provides insight into the acquisition of genes characteristic of an obligate intracellular lifestyle. J Bacteriol 194:376–394. doi:10.1128/JB.06244-11 22056929 PMC3256634

[26] Crooks GE, Hon G, Chandonia J-M, Brenner SE. 2004. WebLogo: a sequence logo generator. Genome Res 14:1188–1190. doi:10.1101/gr.849004 15173120 PMC419797

[27] Edgar RC. 2004. MUSCLE: multiple sequence alignment with high accuracy and high throughput. Nucleic Acids Res 32:1792–1797. doi:10.1093/nar/gkh340 15034147 PMC390337

[28] Guillotte ML, Gillespie JJ, Chandler CE, Rahman MS, Ernst RK, Azad AF. 2018. Rickettsia lipid A biosynthesis utilizes the late acyltransferase LpxJ for secondary fatty acid addition. J Bacteriol 200:e00334-18. doi:10.1128/JB.00334-18 30012728 PMC6148475

[29] Pettersen EF, Goddard TD, Huang CC, Couch GS, Greenblatt DM, Meng EC, Ferrin TE. 2004. UCSF Chimera--a visualization system for exploratory research and analysis. J Comput Chem 25:1605–1612. doi:10.1002/jcc.20084 15264254

[30] Dovala D, Rath CM, Hu Q, Sawyer WS, Shia S, Elling RA, Knapp MS, Metzger LE. 2016. Structure-guided enzymology of the lipid a acyltransferase LpxM reveals a dual activity mechanism. Proc Natl Acad Sci U S A 113:E6064–E6071. doi:10.1073/pnas.1610746113 27681620 PMC5068295

