# Supplementary material for: Structural determination of Rickettsia lipid A without chemical extraction confirms shorter acyl chains in later-evolving spotted fever group pathogens
Source: mSphere. 2024 Jan 23;9(2):e00609-23. doi: 10.1128/msphere.00609-23 (PMC10900879; doi:10.1128/msphere.00609-23)
Supplement: Fig. S1 — The Raetz pathway for Rickettsia lipid A biosynthesis is variable for 2′ secondary acyl chain addition. [file msphere.00609-23-s0001.pdf]

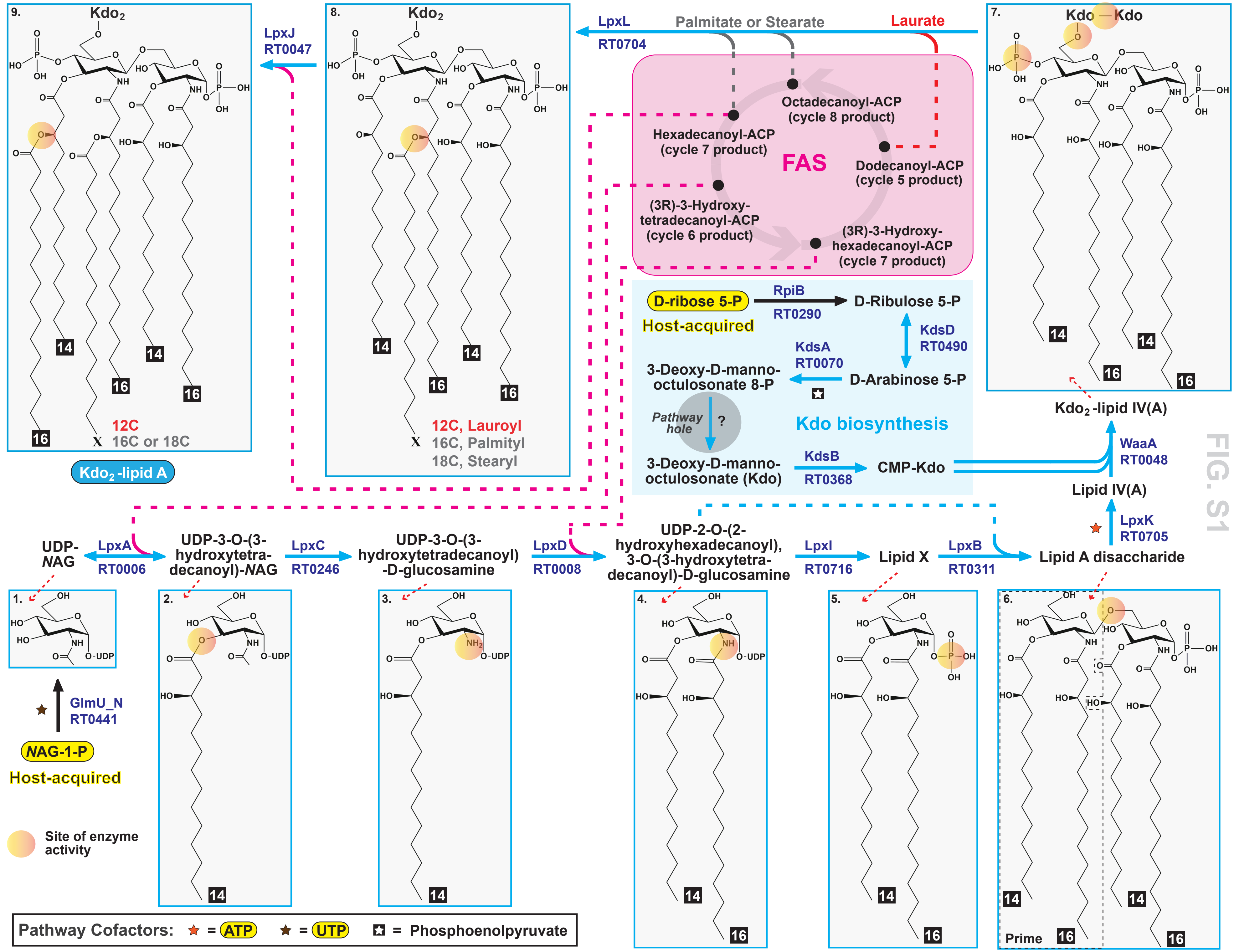

9.
Kdo2
Kdo2 -lipid A
12C
16C or 18C
LpxJ
RT0047
8.
12C, Lauroyl
16C, Palmityl
18C, Stearyl
LpxL
RT0704
Palmitate or Stearate
Laurate
7.
Kdo — Kdo
Octadecanoyl-ACP
(cycle 8 product)
Hexadecanoyl-ACP
(cycle 7 product)
FAS
Dodecanoyl-ACP
(cycle 5 product)
(3R)-3-Hydroxy-
tetradecanoyl-ACP
(cycle 6 product)
(3R)-3-Hydroxy-
hexadecanoyl-ACP
(cycle 7 product)
D-ribose 5-P
Host-acquired
RpiB
RT0290
D-Ribulose 5-P
KdsD
RT0490
KdsA
RT0070
3-Deoxy-D-manno-
octulosonate 8-P
D-Arabinose 5-P
Pathway hole
?
Kdo biosynthesis
3-Deoxy-D-manno-
octulosonate (Kdo)
KdsB
RT0368
CMP-Kdo
Kdo2 -lipid IV(A)
WaaA
RT0048
Lipid IV(A)
LpxK
RT0705
UDP-NAG
LpxA
RT0006
UDP-3-O-(3-hydroxytetra-decanoyl)-NAG
LpxC
RT0246
UDP-3-O-(3-hydroxytetradecanoyl)-D-glucosamine
LpxD
RT0008
UDP-2-O-(2-hydroxyhexadecanoyl),
3-O-(3-hydroxytetra-decanoyl)-D-glucosamine
LpxI
RT0716
Lipid X
LpxB
RT0311
Lipid A disaccharide
1.
2.
3.
4.
5.
6.
Prime
GlmU_N
RT0441
NAG-1-P
Host-acquired
Site of enzyme activity
Pathway Cofactors: = ATP = UTP = Phosphoenolpyruvate

FIG. S1

FIGURE S1. The Raetz pathway for *Rickettsia* lipid A biosynthesis is variable for 2' secondary acyl chain addition. As *Rickettsia* species lack enzymes for synthesizing amino sugars and pentose phosphates, they are predicted to use host *N*-acetylglucosamine-1-P and ribose-5-P to initiate biosynthesis of lipid IV(A) and 3-deoxy-D-manno-octulosonate (Kdo), respectively (3, 6). The enzymes synthesizing these two molecules (GlmU_N, LpxA, LpxC, LpxD, LpxI, LpxB, LpxK, RpiB, KdsD, KdsA, KdsB, WaaA) are conserved in all *Rickettsia* genomes and ultimately generate Kdo_2-lipid IV(A). Note: Kdo_2 is not shown on the final structure as its structure, as well as structures for inner and outer core oligosaccharide, have not been determined for rickettsiae. Variable 2' secondary acyl chain addition by LpxL is shown at *top-center*, with lauroyl-Kdo_2-lipid IV(A) synthesized for *R. parkeri* and *R. rickettsii* strains and either palmityl- or stearyl-Kdo_2-lipid IV(A) synthesized for *R. akari*, *R. typhi*, and *R. montanensis*. By contrast, 3' secondary chain acylation is conserved in all generated structures, with palmitic acid added to 3'-hydroxytetradecanoate by the late acyltransferase LpxJ that we previously characterized (30). Enzyme names are accompanied with locus tags for *R. typhi* str. Wilmington.
